# Not Only Metabolic Complications of Childhood Obesity

**DOI:** 10.3390/nu16040539

**Published:** 2024-02-15

**Authors:** Sebastian Ciężki, Emilia Odyjewska, Artur Bossowski, Barbara Głowińska-Olszewska

**Affiliations:** Department of Pediatrics, Endocrinology, and Diabetology with Cardiology Division, Medical University of Bialystok, 15-274 Białystok, Poland

**Keywords:** childhood obesity, obesity complications, non-metabolic complications

## Abstract

The increasing incidence of obesity in the pediatric population requires attention to its serious complications. It turns out that in addition to typical, well-known metabolic complications, obesity as a systemic disease carries the risk of equally serious, although less obvious, non-metabolic complications, such as cardiovascular diseases, polycystic ovary syndrome, chronic kidney disease, asthma, thyroid dysfunction, immunologic and dermatologic conditions, and mental health problems. They can affect almost all systems of the young body and also leave their mark in adulthood. In addition, obesity also contributes to the exacerbation of existing childhood diseases. As a result, children suffering from obesity may have a reduced quality of life, both physically and mentally, and their life expectancy may be shortened. It also turns out that, in the case of obese pregnant girls, the complications of obesity may also affect their unborn children. Therefore, it is extremely important to take all necessary actions to prevent the growing epidemic of obesity in the pediatric population, as well as to treat existing complications of obesity and detect them at an early stage. In summary, physicians treating a child with a systemic disease such as obesity must adopt a holistic approach to treatment.

## 1. Introduction

Over the last few decades, obesity has become a huge challenge for the healthcare system on a global scale [1,2]. The latest WHO report on data collected in 2018–2020 in European countries reported that approximately 29% of children aged 7–9 were overweight or obese, while another showed that this problem affected more than 17% of children worldwide [3].

Obesity and overweight in childhood may result in problems with maintaining a normal body weight in adulthood. It is a complex, multifactorial disease characterized by abnormal or excessive accumulation of fat tissue, which is associated with an increased risk of many serious diseases as well as its premature onset if obesity occurs from an early age. Numerous complications of obesity, previously most often described in relation to adults, such as cardiovascular diseases (CVD), left ventricular hypertrophy, or hypertension, are increasingly observed in children and adolescents. The incidence of these complications increases with the prevalence of obesity itself. Problems affect both physical and mental wellness [4]. Numerous studies have shown the metabolic complications of obesity, which have been widely discussed in previous works, such as insulin resistance, hyperglycemia, dyslipidemia, hypertension, and type 2 diabetes, negatively affect physical health and are even associated with higher overall mortality in the population [5,6,7,8]. The review we present concerns less known and studied non-metabolic complications (Table 1), which at the same time significantly affect the patient’s health, quality of life, and sometimes even lead to disability. 

Recently, the concept of distinguishing between metabolically healthy obesity (MHO) and metabolically unhealthy obesity (MUO) has been developed. MHO is characterized by the absence of cardiometabolic disorders, including insulin resistance, impaired glucose tolerance, dyslipidemia, and hypertension, despite excessive accumulation of adipose tissue. However, this is most likely temporary and the risk of developing cardiometabolic diseases is still higher than in healthy slim people [9]. Moreover, the data collected in our review show that both MHO and MUO can lead to various health-threatening, non-metabolic complications of obesity; therefore, any obesity disease is an indication for weight loss intervention.

In this narrative review, through a comprehensive, critical, and objective analysis of the current literature, we will try to explain how excessive body weight affects the above-mentioned elements and why it is considered a great threat to the health of the circulatory system, metabolism, and psychosocial development of children. The division into metabolic and non-metabolic complications is conventional because everything related to obesity is associated with metabolic disorders and hormonal, cytokine, and inflammatory activity of adipose tissue.

Interventions to prevent and treat childhood obesity are essential to avoid the burden of comorbidities. The complications we describe are equally important from the point of view of both the patient’s health and society. They cause absences from school and less establishing and maintaining contacts with childhood peers, thus reducing development potential. In later life, obesity may be associated with problems in finding a job due to decreased work efficiency and more frequent sickness absence. A poorer social life from an early age may also make it difficult to start a family and give birth to a healthy child.

## 2. Metabolic Complications of Obesity in Children

### 2.1. Insulin Resistance

By definition, insulin resistance (IR) is a state of reduced response to insulin of target tissues with normal or elevated serum insulin levels and is the reciprocal of insulin sensitivity [7]. It is one of the earliest and most serious changes in hormonal balance in obesity, regardless of the age at which overweight occurs, and its prevalence in the pediatric population is estimated at 11.5% (according to some studies, even up to 44%) [10]. The consequence of IR is chronic, compensatory hyperinsulinemia allowing for maintenance of the required biological effect in the form of obtaining proper glucose levels while reducing the suppression of glucose production in the liver [11]. When insulin release is no longer able to compensate for IR, it leads to disorders of carbohydrate metabolism, such as type 2 diabetes mellitus (T2DM). Typical markers of the state of IR are increased levels of insulin, which is mainly responsible for the adverse effects of this phenomenon, glucose, and free fatty acids (FFA) in the bloodstream [12]. The occurrence of IR is primarily associated with visceral adipose tissue and, as a consequence of its excess, abdominal (visceral) obesity. Therefore, a greater degree of IR and metabolic disorders are also observed in children with a greater share of visceral fat in the child’s overall BMI [13]. Progression of decrease in tissue insulin sensitivity may lead not only to T2DM but also to dyslipidemia, metabolic syndrome (MetS), or non-alcoholic fatty liver disease (NAFLD).

### 2.2. Diabetes Mellitus

Until recently, type 2 diabetes was diagnosed almost exclusively in adults, but over the past few decades, the incidence of T2DM among children and adolescents worldwide has increased several fold [14], interestingly coinciding with a parallel increase in childhood obesity [1]. 

The primary pathology here is insulin resistance, impaired glucose tolerance, and hyperinsulinemia [15]. The first detected biochemical abnormalities indicate the development of so-called prediabetes: abnormal fasting glucose or impaired glucose tolerance in the oral glucose tolerance test (OGTT). Sinha et al. found impaired glucose tolerance in 21% of adolescents aged 11–18 and 25% of children aged 4–10, with both groups affected by obesity [16]. Furthermore, children with obesity are four times more likely to develop T2DM compared to children with normal BMI values [17]. Compared to adults with T2DM, in whom secondary complications develop later in life despite long-term insulin resistance, clinical diabetes and its complications develop faster and more aggressively in children. In addition, children show an earlier need for insulin treatment [18].

Obesity is suggested to be associated with an increased incidence of type 1 diabetes mellitus (T1DM) [19]. The “accelerator hypothesis” posits that obesity-induced insulin resistance in predisposed individuals accelerates the development of clinically overt T1DM [20]. Additionally, obesity is also believed to be connected with the evolution of an autoimmune response against multiple organs, together with pancreatic β-cells. Inflammation induced by obesity appears to increase the risk of developing clinical T1DM in the presence of only one type of autoantibodies—islet cell antibodies (ICA) [15]. The appearance of autoimmune response against pancreatic islet antigens is associated with lower insulin sensitivity and occurs more often in obese patients with impaired carbohydrate metabolism [21]. Moreover, long before T1DM, obesity is clinically evident and increases the switch to two autoantibodies in childhood and adolescence, although only in specific genetic groups [22]. Nevertheless, our previous study showed a significant decrease in C-peptide concentration, hence a deterioration in residual β-cell function within two years of diagnosis in children with the highest BMI, which may suggest a negative impact of obesity on the course of T1DM and the duration of partial remission [23].

### 2.3. Dyslipidemia

In the general pediatric population, the incidence of dyslipidemia is estimated at 8–20% [24], while data show that, in the population of children with obesity, abnormal lipid values range from 20% to over 40% [25,26]. The paper by Nielsen et al. showed that the risk of lipid disorders is 2.8 times higher in children with BMI > 90th percentile than in children with body weights within normal limits [27]. Obesity-linked dyslipidemia is primarily characterized by elevated plasma FFA and triglyceride levels, decreased high-density lipoprotein (HDL) levels, and abnormal low-density lipoprotein (LDL) composition [28]. Elevated FFA levels appear to play a significant role as a cause of insulin resistance. It has been suggested that increased levels of FFA and intracellular lipids inhibit the insulin signaling mechanism, leading to reduced glucose transport into muscle [10]. FFAs also play a mediating role between insulin resistance and β-cell dysfunction, indicating that reducing FFA levels may be a desirable therapeutic target [29].

Adipose tissue directly drives the development of many risk factors through its endocrine role of secreting inflammatory cytokines, proteins of the renin-angiotensin system, or metabolizing sex steroids and glucocorticoids [30]. It also influences reduced secretion of adiponectin—an adipose tissue-specific circulating protein known to be a homeostatic factor for regulating glucose levels and lipid metabolism as well as an insulin-sensitizing hormone affecting a wide range of tissues [6]. 

The body fat mass and body mass index have emerged to be the most important variables that determine circulating leptin concentrations. Obesity is associated with increased levels of leptin, which has only very limited effects due to tissue resistance to leptin associated with insulin resistance and abdominal obesity. In addition to the regulatory role of leptin in energy intake and expenditure, the independent involvement of leptin in maintaining insulin and glucose homeostasis has been documented. Leptin inhibits insulin secretion from the pancreas and mobilizes peripheral organs to increase glucose uptake. It follows that both hypothalamic leptin deficiency due to leptinopenia and reduced leptin transport across the blood–brain barrier due to obesity-related hyperleptinemia precede the pathophysiological consequences of diabetes types 1 and 2 [31]. 

### 2.4. Hypertension

Obesity is the most common risk factor for hypertension in today’s children. As many as 39% of those with severe obesity, 26% of children affected by obesity, and 20% of minors who are overweight suffer from hypertension, but its prevalence among children of normal body weight is only 5% [32]. Being overweight especially increases systolic blood pressure, and this relationship is even stronger in children than in adults. Systolic blood pressure in children is highly dependent on BMI, skinfold thickness, and waist circumference, and it increases with these variables [33]. Additionally, previously mentioned leptin, which is excessively secreted from adipose tissue, causes systemic vasoconstriction, sodium retention, and also affects the synthesis of nitric oxide and activation of the sympathetic nervous system, which leads to an increase in blood pressure [34].

### 2.5. Metabolic Syndrome

Metabolic syndrome has not yet been assigned a specific definition. Notwithstanding, it is generally considered as a coexistence of abdominal obesity, high insulin resistance, hyperglycemia, atherogenic dyslipidemia, and elevated blood pressure [35]. Reinehr et al. compared different definitions of MetS in a cohort of 1205 children and adolescents and found a wide range of prevalence ranging from 6 to 39%, depending on the proposed definition [36]. Therefore, there is a crucial need to establish a detailed definition and diagnostic criteria of MetS for the pediatric population in order to enable prevention, early detection, and treatment of this condition. 

Even though MetS manifests itself more often in people with diabetes than in the general population, a noticeably higher prevalence of its criteria was identified in the group of obese and overweight patients in comparison to patients with a normal body weight range [37]. Many studies have identified a sedentary lifestyle and unbalanced dietary patterns resulting in excess body weight as the main pathological factors inducing risk factors belonging to the MetS cluster [35]. 

By far the most effective strategy for preventing the occurrence of MetS in the future is to proactively prevent obesity among children and adolescents. MetS significantly increases the risk of CVD and other mortality causes, like kidney failure, by 1.5–2-fold in the pediatric age group [35]. Identifying children at risk of developing MetS could enable pediatricians to quickly recognize and treat children and remains an important task that may contribute to reducing the incidence of cardiovascular disease and T2DM in later life [38]. This involves the need for multi-aspect medical intervention, both non-pharmacological and often pharmacological. Moreover, MetS is also associated with other disease processes associated with childhood obesity, including NAFLD [39] and renal dysfunction, with implications for chronic kidney disease [40].

### 2.6. NAFLD/MAFLD

Abdominal obesity (especially a high percentage of visceral fat accumulation and increased waist circumference), insulin resistance, and hypertension are typical features of children with NAFLD—the presence of hepatic steatosis [41]—which is considered as the most common liver disease during childhood [42]. The pathogenesis of pediatric NAFLD is not fully elucidated; however, several studies have reported elevation of BMI as an independent risk factor for fatty liver disease [43,44,45]. An excessive amount of energy is routinely stored in adipose tissue, causing adipocyte hyperplasia; unfortunately, if the buffering capacity of adipose tissue is impaired, ectopic fat aggregation may occur in various organs, along with the liver [46]. Results from a cohort of obese Taiwanese children showed that BMI z-score independently predicted both the occurrence and remission of NAFLD. Individuals with an increased BMI had a higher risk of developing NAFLD, and those with a lower BMI had a higher chance of NAFLD remission [44]. Moreover, a study conducted on obese Danish children showed that a 10% weight reduction was correlated with a reduction in liver steatosis (assessed by ultrasound) by almost 45–30% [47], while a different study showed that, after a year of weight loss, serum ALT levels were significantly reduced and the incidence of fatty liver disease decreased from 100% to 33% [48]. 

NAFLD can present in three stages of severity, namely steatosis, non-alcoholic steatohepatitis (NASH), where the accumulation of toxic fat in the steatosis stage is accompanied by inflammation, and finally cirrhosis, where liver damage begins to cause fibrosis leading to organ failure. NAFLD in children is associated with hepatic and extrahepatic morbidity and mortality. Children with NAFLD often have comorbidities such as CVD, obstructive sleep apnea, polycystic ovary syndrome (PCOS), and osteopenia [49,50]. Studies show that weight gain in childhood and adolescence is associated with an increased risk of not only NAFLD but also end-stage liver disease and hepatocellular carcinoma later in life [51,52]. 

Therefore, it is extremely important for us to evaluate NAFLD in children because it may cause significant adverse effects. Body weight control may have a beneficial effect on the course of this disease. There is some evidence that a low-carbohydrate and low-glycemic index diet may improve hepatic steatosis and fibrosis [53].

A new terminology for NAFLD has recently been updated based on international consensus—metabolic (dysfunction) associated fatty liver disease (MAFLD) [54]. The global pervasiveness of MAFLD, estimated from existing fatty liver disease data, is 33.78% in the general pediatric population and 44.94% in obesity clinics [55]. The diagnosis of MAFLD is based on the accumulation of fat in the liver and one of the following three criteria: overweight/obesity, T2DM, or symptoms of metabolic dysregulation [56].

## 3. Non-Metabolic Complications of Obesity in Children

### 3.1. Cardiovascular Consequences

The important contributions of intra-abdominal adiposity (IAA) to dyslipidemia and insulin resistance provide an indirect, though clinically important, link to the genesis and progression of atherosclerosis and cardiovascular diseases [57]. Excess fat tissue is usually accompanied by elevated levels of high-sensitivity C-reactive protein (hsCRP), a marker of inflammation [58]. Inflammation is triggered by immune cells that attack dysfunctional fat tissue. As obesity progresses, inflammatory cells infiltrate the adipose tissue, pancreas, and other tissues. The obesity-related phase of atherosclerotic inflammation causes coronary artery calcification [59]. Additionally, obesity itself in young children is associated with endothelial cell dysfunction [60] and increased carotid intima media thickness (c-IMT) [61].

There is a strong link between childhood obesity and CVD in adulthood. However, normalization of weight status before puberty appears to eliminate this risk.

### 3.2. Cardiomyopathy

A recent prospective cohort study of 1.7 million subjects showed that an increased BMI in adolescence is associated with the development of cardiomyopathy in adulthood and an up to 8-fold increase in the risk of dilated cardiomyopathy if BMI ≥ 35 kg/m^2^ [62]. The severity and duration of obesity are considered important factors affecting changes in cardiac performance and morphology. Multiple mechanisms determine the progression of myocardial remodeling in people with obesity, including hemodynamic changes such as cardiac output and increased blood volume, leading to left ventricular hypertrophy and dilation [63]. Abnormal lipid metabolism, including excessive activity of adipocyte cell signaling molecules, as well as oxidative stress and inflammation, can potentially cause cardiac fibrosis and impaired systolic and diastolic function [64]. The manifestation of particular types of cardiomyopathy is related to body weight, although it is strongest in the case of dilated cardiomyopathy [62]. These findings are disturbing due to the high mortality rate of patients with this type of cardiomyopathy [65].

### 3.3. Endocrine and Gynecological Consequences

The growing incidence of obesity in the childhood population carries the risk of endocrine and gynecological consequences such as central precocious puberty [66], earlier attainment of pubertal milestones [67], menstrual disturbances [68], the occurrence of polycystic ovary syndrome [66], and alterations in thyroid hormonal status [69]. 

#### 3.3.1. Precocious Puberty

Recently, an interesting link between overweight and the occurrence of central precocious puberty (CPP) has been noted, particularly in girls [70]. These findings appear to be supported by a Chinese population-based study by Liu et al., which found that the incidence of precocious puberty was higher among obese (48.00%) and overweight (27.94%) girls compared to their normal-weight peers (8.73%) [71]. In turn, among boys, precocious puberty prevalence was greater in the group of obese subjects (6.78%) than in the normal-weight group (2.86%) [71]. Interestingly, the overweight group of boys presented lower prevalence of precocious puberty than the normal-weight group (*p* > 0.05) [71]. Thus, obesity rather than overweight has been shown to be a risk factor for the occurrence of precocious puberty in boys [72]. In addition, a study conducted on a group of Chinese children by Chen et al. also demonstrated that obesity has an apparent impact on the occurrence of precocious puberty in both sexes [73]. What is more, reduction in BMI-SDS has been previously shown to be related to earlier gonadotropin-dependent onset of puberty in boys and delayed attainment of puberty in girls, which may imply that girls dealing with obesity experience earlier puberty and boys with obesity demonstrate delayed puberty [74]. It is important to remember that children with excessive weight are more likely to present isolated, mild manifestations of precocious puberty, such as precocious pubarche (the most common form), axillarche, and thelarche [68]. It appears that precocious pubarche and thelarche may result in a modest acceleration of skeletal growth and maturation [75,76]. The results of a Chinese study indicated that the bone age of girls aged 4–8 years with isolated premature thelarche may be substantially advanced [77]. Moreover, in the same group of subjects, BMI-SDS, serum IGF-1 SDS, and DHEAS SDS were identified as factors associated with an increased risk for advanced bone age [77]. The majority of sources in the literature assert that these mild forms of precocious puberty are self-limited [76] and usually do not need any treatment [68,78]. Nevertheless, follow-up is recommended [79] as they could potentially be a manifestation of central precocious puberty that may manifest in later years [76]. However, taking the above into account, further research into the treatment of mild forms of precocious puberty and their implications is needed.

There are several possible explanations for the cause of precocious puberty in children with obesity. Firstly, it may be a result of increased levels of leptin (a hormone secreted by adipocytes that is a permissive factor in the onset and progression of puberty) in both obese genders and kisspeptin (a peptide that is crucial in the initiation and development of puberty) in girls suffering from obesity [80]. Furthermore, in the increased amount of adipose tissue in the course of obesity occurs an intense aromatization of adrenal and ovarian androgens to estrogen, which is reflected in earlier adrenarche and thelarche in girls [81]. In addition, it was shown that obese prepubertal girls had significantly higher BMI-SDS, leptin, insulin, and HOMA-IR values than the control group, and that girls with obesity in early puberty presented higher BMI-SDS, leptin, IGF-1, IGFBP3, insulin, HOMA-IR, LH, LH/FSH ratio, ACTH, DHEA-S, androstenedione, testosterone, and free testosterone values than the control group [82]. Consequently, the hyperinsulinemia that occurs in girls experiencing obesity is considered to play a facilitating role in early puberty by leading to stimulation of adrenal and ovarian androgen production, decreased hepatic synthesis of sex hormone-binding globulin (SHBG), and increased aromatase activity in adipocytes [80,83,84]. It was observed that an excess of insulin is connected with the occurrence of precocious pubarche [68]. Furthermore, stimulation of adrenal androgen production by hyperinsulinemia may present in the form of precocious pubic and axillary hair appearance before the age of 8 years in girls and 9 in boys [68]. It may occur together with mild acne, pubertal sweat odor, and moderately accelerated growth and bone age [68].

Interestingly, previous studies have shown that earlier puberty may be a consequence of early adiposity rebound [85]. Throughout a child’s development, typically there is an initial rise in BMI during the first year of life, followed by its decline, reaching a nadir around the age of 6 [85]. Thereafter, an increase in BMI occurs again, which is commonly known as adiposity rebound (AR) [85]. In their study, German et al. demonstrated that, among children who experienced an earlier adiposity rebound, boys exhibited earlier pubarche and faster pubertal progression, while girls showed an earlier ages of menarche and thelarche compared to their peers who underwent AR later [86]. Furthermore, evidence suggests that early adiposity rebound is a contributing factor to the increased risk of polycystic ovary syndrome (PCOS) and elevated BMI in adulthood [87]. Very early (<42 months) and early (42–59 months) AR have also been shown to be associated with the deterioration of cardiac and metabolic parameters, including lipid parameters, total and central adiposity, blood pressure, and HOMA-IR, in children at the age of 10 years [88].

It turns out that excessive body weight not only leads to the emergence of precocious puberty in children but also to the earlier achievement of pubertal milestones. It was found that both girls and boys with higher BMI reached all of the pubertal milestones earlier, in a dose-dependent manner [67]. Moreover, it has been demonstrated that overweight and obesity contribute to earlier breast development and menarche in females, which can have detrimental effects on their mental health, social functioning, and physical health in adulthood, like increased risk of developing type 2 diabetes mellitus, cardiovascular diseases, shorter adult height, or post-menopausal breast cancer [70,80]. The results of the study by Li et al. are in line with these findings and clearly indicate that higher BMI before puberty is connected with earlier breast development and the appearance of menstruation in girls and with earlier first spermatorrhea (which indicates late-stage puberty in boys) and testicular development among boys [89]. Interestingly, studies are inconclusive regarding the impact of overweight and obesity on puberty in boys. Some findings show that overweight boys reach puberty faster, while boys with obesity experience a delay in pubertal development [90]. The results of the study by Lee et al. demonstrated that boys dealing with overweight experienced early puberty for Tanner scale GD2 or above compared to their normal-weight peers (9.3 years for overweight vs. 10.0 years for normal-weight boys) and Tanner GD5 or above (14.5 years for overweight vs. 15.2 years for normal-weight boys). Boys suffering from obesity were shown to have delayed puberty for GD5 and above compared to their overweight and normal-weight counterparts (15.4 years for obese vs. 14.5 years for overweight and 15.2 years for normal-weight boys) [90]. A potential explanation for such correlations may be that adipose tissue-derived aromatase activity can lead to excessive estrogen production in boys, and it is hypothesized that increased estrogen production inhibits puberty in boys with obesity, while no such effect occurs in those who are overweight [90]. Nevertheless, the influence of overweight and obesity on puberty timing in boys as well as the mechanisms that affect this process require further investigation.

It is worth adding that, among girls experiencing idiopathic precocious puberty, the pattern of constitutional advancement of growth is commonly observed [91]. It is associated with early puberty and marked by accelerated early-stage growth, peaking in the initial 2–4 years of life [92]. Intriguingly, this pattern is also correlated with an increased incidence of childhood obesity, and the precise causes of this condition are not yet known [92]. Furthermore, in a multicohort study conducted on American children, it was observed that girls who experienced an accelerated BMI gain in late infancy tended to reach menarche earlier, whereas those with rapid BMI growth during early childhood achieved pubic hair stage greater than 1 more quickly [93]. Therefore, the occurrence of accelerated growth during the first few years of life can help identify children who are likely to start puberty earlier [93].

Remarkably, insulin-like growth factor-1 (IGF-1) has been identified as a very prominent factor accelerating the onset of puberty through its inhibitory effect on dynorphin, a neuropeptide that suppresses the pre-pubertal secretion of GnRH [94]. Findings suggest that IGF-1 may also contribute to the occurrence of a pattern of constitutional advancement of growth and puberty (CAGP), as IGF-1 has been found to be increased in this condition [94]. This pattern is more typically seen in girls [94]. It appears that, in the course of obesity, there occurs an increase in free IGF-1 levels, predominantly through a decrease in IGF-1-binding protein levels caused by obesity-related hyperinsulinemia [95]. On the other hand, GH secretion in individuals with obesity is reduced [95]. Interestingly, greater levels of IGF-1 before puberty were demonstrated to be associated with an earlier onset of thelarche and menarche among girls [96]. Similarly, higher growth before puberty was linked to lower ages of thelarche and menarche [96]. In the case of boys, higher pre-pubertal IGF-1 levels were associated with earlier gonadarche and increased height [96]. A German study found that children with obesity showed higher growth at birth than their normal-weight counterparts and had an accelerated growth rate thereafter, which was associated with increased levels of IGF-1, insulin, and leptin in these children [97]. By contrast, during puberty, children suffering from obesity exhibited a decline in growth rate, which was correlated with decreases in IGF-1 and testosterone levels in boys and estradiol levels in girls [97]. The exact mechanism for the drop in IGF-1 levels during puberty remains unclear; however, it is hypothesized that low testosterone levels in boys with obesity may be a contributing factor, although this hypothesis does not seem to apply to girls [97]. In the same study, it was observed that, during the onset of puberty, boys struggling with obesity were 6.6 months older while girls affected by obesity were 9.1 months younger than normal-weight subjects [97]. Additionally, menarche occurred 8.9 months earlier in girls experiencing obesity compared to their normal-weight peers [97].

What is more, adolescents who are overweight or obese have been shown to be more likely to experience gynecological and obstetric complications, both in adolescence and adulthood [81]. An interesting link has been found between obesity and menstrual disorders, such as irregular and infrequent menstrual cycles or even amenorrhea, lack of ovulation, PCOS, and heavy menstrual bleeding in both adolescence and adulthood [80]. Recent studies indicate that having a higher BMI in childhood is related to menstrual irregularity, and that a greater childhood BMI and waist/height ratio (WHtR) is associated with the development of PCOS in adulthood among white but not black participants [98]. Moreover, pregnant adolescents with obesity are predisposed to the occurrence of perinatal complications such as preeclampsia, gestational hypertension, gestational diabetes, cesarean and instrumental delivery, and induction of labor [81]. For instance, the results of a recent study by Ambia et al. showed that young adolescents struggling with obesity (≤15 years old) were at higher risk of developing pre-eclampsia and delivering by cesarean section than those who were non-obese [99]. The prevalence of overweight and obesity in childhood and adolescence may also lead to macromastia and a greater likelihood of developing breast and endometrial cancer [81]. 

#### 3.3.2. Polycystic Ovary Syndrome

As mentioned earlier, obesity in girls contributes to a greater likelihood of developing PCOS. It should be added that PCOS is the most frequent endocrinopathy in young adult females and the number one cause of non-ovulatory infertility [80]. The diagnosis of PCOS in adolescent girls can be made after meeting both criteria: (1) the occurrence of menstrual disturbances (irregular menstruation, oligomenorrhea, or secondary amenorrhea) and (2) the presence of clinical (in the form of hirsutism or moderate to severe acne) or biochemical hyperandrogenism [68,100]. It is challenging to diagnose PCOS in adolescent girls, as the key features of a PCOS diagnosis, like irregular menstrual cycles, acne, and polycystic ovarian morphology on pelvic ultrasound, coincide with the physiological changes seen during puberty [100]. Furthermore, it was demonstrated that PCOS coexists with a number of metabolic disorders, such as the occurrence of both insulin resistance and hyperinsulinemia [101]. Also, adolescents with PCOS are widely overweight and obese, and they more often develop metabolic syndrome, impaired glucose tolerance, and type 2 diabetes, so patients with PCOS should undergo screening for the presence of these conditions [101]. According to a meta-analysis describing the impact of obesity on metabolic disturbances in adolescent PCOS, adolescents with obesity and PCOS exhibited significantly reduced levels of SHBG and HDL-C and substantially elevated levels of triglycerides, leptin, fasting insulin, LDL-C, and free testosterone when compared to normal-weight adolescents with PCOS [102]. In addition, they showed significantly elevated fasting insulin, LDL-C, free testosterone, and 2 h OGTT levels in comparison to non-PCOS adolescents affected by obesity [102]. It has been demonstrated that the underlying cause of PCOS in adolescents with obesity actually lies in hyperinsulinemia, which, as mentioned earlier is able to promote androgen production in the ovaries and adrenal glands as well as decrease the hepatic synthesis of sex hormone-binding globulin (SHBG), thus leading to an excessive amount of androgens [68]. The interesting study by Jobira et al. indicated that there exists an alteration in the composition of the intestinal microbiota among adolescents experiencing PCOS and obesity, although further investigation is needed to understand the relationship between the microbiome and androgens, which has the potential to provide a new therapeutic pathway [64].

#### 3.3.3. Thyroid Function in Childhood Obesity

Recently, there has been increased medical interest in thyroid function in obesity [69]. Individuals with obesity often exhibit alterations in thyroid function and thyroid structure, which is similar to that of Hashimoto thyroiditis, yet in the absence of disease [103]. It was previously demonstrated that TSH and total T4 levels were substantially elevated in children affected by obesity in comparison to their non-obese peers, and that subclinical hypothyroidism was significantly more prevalent in children with obesity (14%) than in healthy individuals (6.8%) [69]. These findings seem to be in line with the results of the study by Rumińska et al., which revealed that children and teenagers dealing with obesity presented higher TSH levels, even when they were within the normal range compared to normal-weight group, and the concentration of TSH was positively correlated with parameters characteristic of abdominal obesity [104]. However, a study conducted by Krause et al. found that obesity in the pediatric population is linked to increased TSH levels, decreased FT4 concentrations, and higher incidence of abnormally elevated TSH levels [105]. Also, moderate elevation of total T3 and FT3 levels (within the upper normal range or slightly above) is also reported as a hormonal impairment in childhood obesity [106]. Interestingly, a relevant association between the occurrence of hepatic steatosis and TSH levels in children and adolescents experiencing obesity has been also demonstrated [107]. Licenziati et al., in their study, showed that changes in thyroid function and structure in children with obesity tend to be reversed after weight loss [103].

Subclinical hypothyroidism, with a prevalence of up to 10–23% in the obese pediatric population, is a consequence rather than a causative factor of increased body weight [108]. While its mild form is relatively benign (if not connected to Hashimoto’s thyroiditis) in the pediatric population, it has been shown that isolated elevation of TSH levels may be related to an increased risk of developing or progressing cardiovascular disease and non-alcoholic fatty liver disease (NAFLD) in children and adolescents suffering from obesity [109]. Furthermore, it has been also demonstrated that overweight and obesity in children carry a risk of progression from subclinical to overt hypothyroidism [110]. In spite of this, there is limited information regarding the treatment of thyroid disorders co-occurring with obesity in children [109]. It is established that children with subclinical hypothyroidism and serum TSH levels above 10 mU/L are treated, regardless of the underlying cause [108]. However, for lower concentrations, the decision to initiate treatment must be individualized [108]. As obesity is a potentially reversible factor contributing to thyroid dysfunction, the emphasis should be set on diet and changes in lifestyle [111]. It is advisable to reassess thyroid function after losing weight [111] because previous reports indicate that weight loss might lead to the normalization of TSH levels [69,108]. Treatment of subclinical hypothyroidism with levothyroxine has not been proven to contribute to weight loss or normalization of lipid and thyroid parameters in children dealing with obesity [109,110]. Thus, if there are no clinical and laboratory indications of overt hypothyroidism, levothyroxine treatment is not required in such a group of patients [110].

There are several hypotheses that could potentially explain changes in thyroid endocrine status. Firstly, it may originate from a disruption in the hypothalamic-pituitary axis, and it is thought that leptin may be responsible for the alteration of hypothalamic thyrotropin-releasing hormone (TRH) production [112]. Secondly, thyroid hormone resistance caused by a decrease in the amount of TSH or peripheral thyroid hormone receptors may also be one of the explanations for elevated TSH and T3 levels [112]. Furthermore, it was demonstrated that individuals with obesity secrete a high amount of inflammatory cytokines, which could negatively affect the function of the sodium/iodide symporter, ultimately leading to compensatory rise in TSH levels [103]. Finally, thyroid disorders may be the consequence of an adaptation process aimed at increasing energy expenditure [112]. 

In conclusion, the incidence of obesity in the pediatric population raises the risk of developing endocrine and gynecological disorders, which may also have negative consequences on their adulthood health, but the underlying causes of these disturbances are not fully understood and require further research.

#### 3.3.4. Obesity-Induced Hypogonadism

Adult males who are obese present an increased risk of developing hypogonadism compared to those of normal weight [113]. However, the data indicate that the association between obesity and hypogonadism in children and adolescents is less well studied [113]. Nevertheless, the incidence of hypogonadism in male adolescents is on the rise in parallel with the increasing prevalence of obesity [113]. The diagnosis of hypogonadism in boys can be difficult, and symptoms may not appear until puberty [114]. At that time, the development of sexual characteristics may be absent or interrupted [114]. The interactions between sex hormones, excessive aromatase activity in adipose tissue, hormone production by fat cells, and the presence of inflammatory markers occurring in the course of obesity lead to reduced testosterone production and thus contribute to hypogonadism [113]. It has been shown that normal-weight adolescents have higher levels of free testosterone, total testosterone, FSH, and inhibin B and exhibit lower levels of estradiol than their obese counterparts [115]. Thus, an obesity–hypogonadism relationship has been demonstrated and may be driven by increased estradiol levels and a reduced testosterone/estradiol ratio [115].

### 3.4. Other Non-Metabolic Consequences

It is well known that obesity is a systemic disease that potentially affects almost all organ systems [12]. Therefore, obesity occurring during childhood and adolescence can induce or exacerbate multiple diseases and conditions, including those that were not mentioned above, such as respiratory and neurological complications, oral health disorders, immunologic diseases, and renal, gastrointestinal, musculoskeletal, and dermatologic disturbances [12,68].

#### 3.4.1. Respiratory Complications

##### Asthma

Among children, obesity is associated with an increased risk of developing asthma [116], and this risk is more pronounced in girls than boys [117]. According to a meta-analysis based on 18 articles, children dealing with overweight or obesity had 1.23 and 1.40 times higher risks of asthma, respectively [117]. These results seem to be in line with a meta-analysis of case–control studies by Azizpour et al., which demonstrated that children and adolescents who are overweight or obese are more likely to have asthma (1.64 and 1.92 times, respectively) than their normal weight/underweight peers [118]. Moreover, it has been shown that children who gain weight rapidly during the first 2 years of life are more prone to developing asthma in the first 6 years of life than those who gained less weight at an early age [119]. Interestingly, a recent study by Pavlidou et al. found that, among preschool children, not only the prevalence of overweight and obesity but also the presence of abnormal birth anthropometric measures, such as length, birth weight, and head circumference, may increase the likelihood of developing asthma in preschool-aged children [120]. Therefore, an increase in BMI in infancy may be more important in predicting the occurrence of asthma in childhood than being overweight at any specific age [119]. 

What is more, for children already suffering from asthma, coexisting obesity contributes to worse control of symptoms, more frequent and less treatment-responsive exacerbations, poorer response to inhaled corticosteroids (possibly to systemic corticosteroids as well), and decreased quality of life [116]. In addition, children with asthma and obesity are more likely to be unresponsive to bronchodilators and require controlling medications, but on the other hand, they are better responders to leukotriene receptor inhibitors [121]. Thus, apart from the simple co-occurrence of asthma and obesity in certain children, there is increasing evidence of an “obese asthma” phenotype, where increased body weight influences and alters asthma features [121]. In contrast to the “classic” asthma phenotype, the “obese asthma” phenotype can be identified by a low Th2 profile with prevalent infiltration of neutrophils in the bronchial mucosa along with poor infiltration of eosinophils and low levels of IgE [122]. Taken together, these differences in inflammatory response between asthma phenotypes may consequently account for the poorer response to typical asthma treatment in individuals with obesity [123]. 

**Figure 1:**
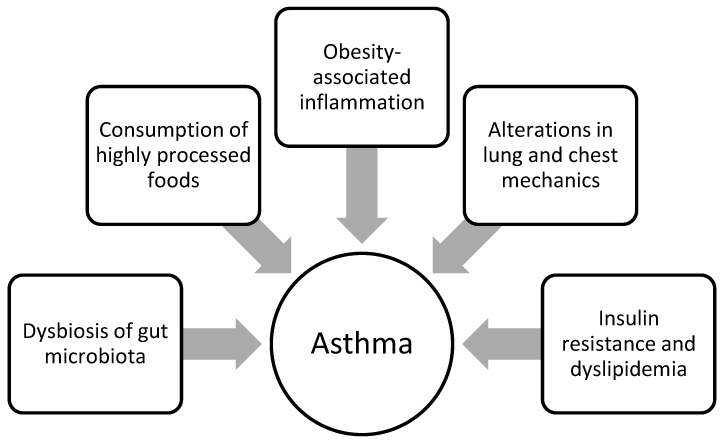
Obesity-related factors contributing to the development of asthma or worsening control of pre-existing disease in children with obesity.

There are several mechanisms that may potentially account for the association between childhood obesity and asthma, such as immunological, mechanical [124], dietary, and metabolic factors or changes in the gut microbiota [116] (Figure 1).

As excessive amounts of adipose tissue in the course of obesity are associated with increased secretion of pro-inflammatory cytokines and leptin and decreased secretion of the anti-inflammatory adipokine, adiponectin [125], it has been demonstrated that pro-inflammatory factors may act as a mediator in the relationship between obesity and the development of asthma [122]. The study by Ma et al. discovered that children with asthma in the observation group with a BMI > 28 kg/m^2^ presented substantially elevated leptin levels and, at the same time, significantly lower adiponectin levels in comparison to their counterparts with a normal BMI. Furthermore, the number of children with severe disease was greater in the observation group, which may indicate that BMI is associated with greater disease severity. 

Obesity has also been shown to adversely affect lung and chest mechanics, as well as cause a reduction in lung compliance [125]. The fat load on the chest wall and abdomen in turn reduces the mobility of the thorax and diaphragm, which ultimately leads to reduced respiratory compliance and increased breathing effort [126]. A meta-analysis that assessed the association between overweight or obesity and pulmonary function in both children and adults revealed that all lung function parameters were reduced in individuals suffering from obesity; however, adults with obesity exhibited lower FEV1 (forced expiratory volume in 1 s), FVC (forced vital capacity), TLC (total lung capacity), and RV (residual volume), whereas children affected by obesity were characterized more by a reduction in FEV1/FVC with intact FEV1 or FVC [127]. This may be related to the finding that obesity in children is associated with airway dysanapsis, defined as a mismatch between lung and airway growth, and more specifically in a faster increase in lung volume and airway length and a concomitant slower increase in airway caliber [128]. Airway dysanapsis associated with obesity is related to adverse clinical outcomes for children with asthma that include a higher risk of severe asthma exacerbations requiring the use of systemic corticosteroids and, in part, their worsened response to treatment [128].

Metabolic dysregulation in the form of insulin resistance and dyslipidemia has previously been shown to likely contribute to asthma in children suffering from obesity [116]. A recent study of children from two independent cohorts, the Tucson Children’s Respiratory Study (TCRS) and the Avon Longitudinal Study of Parents and Children (ALSPAC), found that elevated serum insulin levels in early childhood correspond with higher risks of developing asthma in childhood, adolescence, and adulthood, regardless of BMI, although the processes explaining this phenomenon have not been fully explained [129]. A cross-sectional study aimed at examining the association between obesity, insulin sensitivity, metabolic syndrome, and lung function among American adolescents found that insulin resistance was negatively correlated with FEV1 and FVC in subjects with and without asthma [130]. In addition, it was demonstrated that, among Korean adolescents, subjects with high serum total cholesterol (TC) levels and a high triglyceride (TG)/high-density lipoprotein cholesterol (HDL-C) ratio presented a higher prevalence of asthma [131]. It is likely that dyslipidemia, by activating innate and acquired immunity and enhancing airway inflammatory pathway signaling, consequently increases bronchial smooth muscle tone and airway inflammation and hyperresponsiveness [132].

As a parallel increase in the occurrence of asthma and consumption of highly processed foods, which are poor in antioxidants and rich in saturated fatty acids, has been observed, this may indicate that nutritional habits could play an important role in the development of asthma [133]. Interestingly, a cross-sectional observational study by Tobias et al. found that poor nutritional status (which was characterized by low levels of carotenoids and *n*-3 fatty acids) in adolescent asthmatics with obesity was correlated with airway obstruction and metabolic disturbances [134]. These findings may indicate that carotenoids (antioxidants that are mainly found in fruits and vegetables and have the potential to reduce the risk of many chronic diseases, including asthma) and *n*-3 PUFA (polyunsaturated fatty acids with anti-inflammatory properties) may protect against airway obstruction in adolescents with asthma related to obesity [134]. Furthermore, obese asthmatic children with low vitamin D levels showed worsened lung function in the form of reduced FEV1 and FRC (functional residual capacity) than their normal-weight but also asthmatic counterparts [135]. Hence, changes in diet can induce beneficial effects on obesity-related immune and metabolic impairments that concomitantly contribute to the development of asthma [116]. 

Recent research suggests that the gut microbiota may play a mediating role in the association between obesity and asthma [136]. Dysbiosis of the gut microbiota, which has been observed in both obesity and asthma, negatively affects lipid metabolism and immune response and induces chronic low-grade inflammation. This may indicate that it is a contributing factor in the pathogenesis of obesity-associated asthma, although the underlying mechanisms have not yet been fully investigated [136]. Figure 1 summarises the previously mentioned factors involved in the development of asthma or deterioration of control of pre-existing disease in children affected by obesity.

##### Obstructive Sleep Apnea

Obesity is also a significant factor in the development of sleep-related breathing disorders [137], especially obstructive sleep apnea (OSA), in the pediatric population and it also plays a leading role in the emergence of this condition during adolescence [138]. OSA is manifested by recurrent episodes of partial or complete upper airway obstruction, which disrupts normal sleep and ventilation, eventually causing intermittent hypoxia and/or increased frequency of arousals [139]. The prevalence of OSA in children is estimated to be around 2–3%, with adenotonsillar tissue hypertrophy being the main risk factor determining the development of this condition, especially among young children [140]. However, the rise in the prevalence of obesity in the pediatric population has increased the incidence of OSA to up to 6% of all children [140]. In the course of obesity, fat in the soft tissue of the throat reduces the size of the lumen in the upper airway, leading to increased structural collapse [141]. In addition, increased amounts of fat in the abdominal region decrease lung function, and both of these mechanisms contribute to the occurrence of obstructive sleep apnea [141]. OSA occurring in the pediatric population carries the risk of long-term complications, such as impaired growth and development, hypertension, pulmonary hypertension, endocrine and metabolic disorders, neurocognitive impairment, maxillofacial dysplasia, or a higher risk of cardiovascular episodes if it is not diagnosed and treated in a timely manner [142]. It should be noted that treatment of obesity is associated with improvements in OSA in children and adolescents [143]. A decrease in SDS-BMI has been shown to be strongly associated with a decrease in the apnea-hypopnea index (the sum of the number of apneas and hypopneas divided by the total recording time) after about six months of therapy, indicating that treatment targeting weight loss should be considered as a first-line treatment for OSA in children and adolescents dealing with overweight or obesity [143].

#### 3.4.2. Other Allergic Diseases

In addition to asthma, obesity has been shown to affect the development and course of such allergic diseases as atopic dermatitis, allergic rhinitis, and food allergies [144]. Obesity, due to its inflammatory effects causing deterioration of the epidermal barrier function and changes in the microbiome, has been shown to contribute to the development of atopic dermatitis, worsening of its course, and poorer results of its treatment [144]. On the other hand, the effectiveness of treatment and alleviation of symptoms may be attributed to weight loss [144]. Findings indicate that children with atopic dermatitis demonstrate a greater frequency of being overweight, obese, and dyslipidemic than their healthy peers [145]. According to a meta-analysis by Zhou et al., BMI is associated with an increased likelihood of allergic rhinitis in children rather than adults [146]. The inflammatory milieu often found in obesity or vitamin D deficiency, which increases the risk of atopic diseases, may be responsible for this link [146]. In turn, a Japanese cross-sectional study aimed at examining the relationship between being overweight and the incidence of food allergies among children found a significant correlation between these two conditions. However, this association was observed specifically in girls and not in boys [147]. A possible explanation underlying this relationship may be that obesity and a high-fat diet induce intestinal inflammation, weakening the barrier function of the intestines and leading to diminished immunological tolerance to allergens. Additionally, obesity and a high-fat diet contribute to a shift in the composition of the gut microbiota [144]. It is also worth adding that, concerning another condition, children who are overweight or obese tend to be older at the time of diagnosis of eosinophilic esophagitis than those of normal weight, and abdominal pain is their main symptom [148].

#### 3.4.3. Neurological Complications

##### Pseudotumor Cerebri

Among pubertal children, in contrast to those before puberty, obesity acts as a risk factor for pseudotumor cerebri, a condition in which increased intracranial pressure is observed, although with the absence of a mass [149]. The main symptoms of pseudotumor cerebri include headache, nausea, vomiting, retroocular pain, and visual disturbances [68]; however, it should be noted that the clinical picture in children differs depending on age, and younger children may present less prominent symptoms [150]. Many cases of pseudotumor cerebri are idiopathic, and in such situations the condition is termed primary [151]. Nevertheless, its association with many medications, such as tetracyclines, growth hormones, or retinoids, and medical conditions like cerebral venous abnormalities leads to its classification as secondary [150,151]. A prospective population-based cohort study of British and Irish children with newly diagnosed pseudotumor cerebri syndrome (PTCS) showed that only a minority of cases developed PTCS before the age of 7 years, usually without identified risk factors, whereas the majority of cases occurred from the age of 7 years [152]. The prevalence was twice as frequent in girls and increased with age and degree of overweight [152]. Interestingly, it has been shown that over 80% of PTCS in children aged 12–15 can be attributed to obesity [152]. Potential explanations for the link between obesity and pseudotumor cerebri may be increased intrathoracic and intraabdominal pressures occurring in the course of obesity, which lead to decreased intracranial venous drainage and, consequently, decreased cerebrospinal fluid absorption along with increased intracranial pressure [153]. Also, increased leptin levels in the cerebrospinal fluid of obese patients may account for the occurrence of pseudotumor cerebri in these individuals [153]. Therefore, weight loss is considered to be the only treatment that modifies the course of PTCS [150].

##### Migraine

Additionally, obesity appears to contribute to an increased risk of migraine progression and incidence [68]. The pathogenesis of both obesity and migraine is multifactorial and may be shared between both conditions [154]. It has been shown that neurotransmitters like serotonin, peptides like orexin, inflammatory mediators like calcitonin gene-related protein (CGRP), and adipocytokines such as adiponectin or leptin may have an impact on the common pathophysiology [154]. An Italian study by Tarantino et al. conducted on children and adolescents suffering from migraine found that children who are overweight experienced more frequent migraine attacks than those of normal weight [155]. Thus, the treatment of children affected by obesity and migraine should be focused on weight loss therapies that include exercise, diet, and behavioral interventions [156].

#### 3.4.4. Oral Health Disorders

The incidence of oral health conditions is higher among children and adolescents with obesity [12]. For instance, children experiencing overweight or obesity are more likely to develop dental caries [157]. Obese schoolchildren have been shown to be more likely to develop gingivitis than their normal-weight counterparts, whereas this relationship is modified by gender [158]. It was demonstrated that girls suffering from obesity tend to have a higher likelihood of gingivitis; however, no such relationship was observed among boys [158]. A study of children suffering from both gingivitis and obesity found that obesity amplifies gingivitis [159]. Interestingly, gingivitis through a mechanism involving neutrophil activation can also exacerbate obesity, which is known as disease reciprocity [159]. In a cross-sectional study, it was shown that children and adolescents who were overweight or obese, in contrast to those of normal weight, presented more caries in permanent teeth, increased plaque accumulation and gingivitis, and reduced saliva secretion and buffer capacity [160]. Potential causes linking obesity and dental caries include dietary and lifestyle factors, particularly frequent and excessive consumption of fermentable sugars, changes in saliva composition, and its lower secretion in individuals with obesity [161]. In turn, an impaired immune response and enhanced chronic systemic inflammation may be responsible for the association between obesity and gingivitis, but the underlying factors are not well investigated [158].

On the other hand, studies that aim to investigate the impact of obesity on oral disorders are not entirely consistent. In contrast to the aforementioned results, a cross-sectional study of the pediatric population by Guaré et al. found that individuals experiencing overweight or obesity presented a lower risk of having dental caries, which may be potentially explained by higher levels of protective IgA-s in the saliva of children with excess body mass [162]. These findings seem to be in line with those of the study by García Pérez et al. conducted on Mexican schoolchildren [163]. It has also been shown that, in children aged 6–11 years, BMI was not an independent factor for worse oral hygiene and gingivitis [164]. Taking this into account, the link between obesity and oral diseases should be the subject of further research as evidence is inconclusive.

#### 3.4.5. Immunologic Diseases

The excessive aggregation of adipose tissue present in obesity is associated with subsequent metabolic dysregulation of adipocytes [165]. Adipokines secreted by adipocytes can influence immune cell activity and trigger pro-inflammatory signals, thereby resulting in a chronic, low-grade systemic inflammatory state [165]. Therefore, pediatric obesity has been demonstrated to increase the incidence or severity of immune-mediated or autoimmune diseases, with obesity-related low-grade inflammation as a contributor [12]. 

Interestingly, central obesity in school-aged children emerged as a risk factor for the development of autoimmune diseases, including type 1 diabetes, autoimmune thyroiditis, juvenile idiopathic arthritis, and inflammatory bowel disease in the adolescent period [166].

Moreover, being obese, particularly during adolescence, is commonly related to an increased risk of both adult as well as pediatric multiple sclerosis (MS) [167]. According to the results of a study by Huppke et al. conducted on a group of 453 German children with multiple sclerosis, obesity was associated with a doubled risk of developing MS and a poorer response to first-line treatment [168]. In addition, Harroud et al., in their study, seemed to confirm the association between higher BMI and susceptibility to MS in the pediatric population [169]. It is expected that childhood obesity will contribute to 14% of the total risk of multiple sclerosis in 2035 [170]. 

In our earlier review describing the impact of obesity on type 1 diabetes (T1D) in children, we indicated that excessive body weight and subsequent insulin resistance may contribute to a growing incidence of T1D [171]. Additionally, children who are overweight or obese are more likely to develop complications of diabetes and other resulting diseases compared to normal-weight peers [171]. Having an excessive BMI in adolescence leads to an increased risk of experiencing type 1 diabetes in early adulthood, with 1.54 HR (95% CI 1.23, 1.94) for adolescents dealing with overweight and 2.05 (95% CI 1.58, 2.66) for those with obesity [172].

Notably, studies indicate that children experiencing overweight or obesity have a high prevalence of vitamin D deficiency [173]. They are at risk because obesity has a major impact on the status of this lipophilic vitamin, negatively affecting its concentration [174]. In a study by Tayde et al., children with obesity and those of normal weight were administered the same dose of vitamin D (a single oral dose of 150.000 IU) [175]. Their 25(OH)D levels were assessed one week and one month after administration, revealing that participants suffering from obesity exhibited a 2.2 times poorer increase in 25(OH)D compared to their lean counterparts, both after one week and one month [175]. Therefore, it is suggested that children with obesity might require increased doses of vitamin D or repeated administration of a dose [175]. Vitamin D has been proven to exert a wide variety of immunomodulatory, anti-inflammatory, antioxidant, and antifibrotic functions and has been shown to be deficient in numerous patients with autoimmune diseases [176]. A deficiency in vitamin D concentrations among pediatric patients is associated with a higher susceptibility to immunological conditions, including an increased risk of infections, as it was observed during the COVID-19 pandemic and autoimmune diseases such as type 1 diabetes mellitus and multiple sclerosis [177,178]. Research indicates that vitamin D, during the early stages of life, may provide protection against the development of type 1 diabetes in children genetically susceptible to the disease [179]. This is supported by the observation of lower vitamin D concentrations 18 months prior to the first seroconversion in children with islet autoimmunity having two or more pancreatic antibodies or already existing disease compared to controls who did not show positivity for two or more antibodies [179]. Intriguingly, in Finland, a nation previously marked by a high incidence of vitamin D deficiency, an increase in its concentration among children led to a subsequent plateau in the rising prevalence of type 1 diabetes [180]. 

Taking the above into consideration, it is apparent that a deficiency in vitamin D3 concentrations can play a significant role in the development of children affected by obesity. Therefore, there is urgent need to focus on maintaining its proper serum levels.

#### 3.4.6. Renal Consequences

It was previously demonstrated that children struggling with obesity present a higher risk of chronic kidney disease (CKD) or end-stage renal disease (ESRD) than those having a normal BMI [181]. What is more, obesity in the pediatric population is also a factor that increases the likelihood for disease progression when renal impairment is already present, subsequently raising the mortality risk of children with ESRD [182]. Furthermore, children with obesity may experience a syndrome known as obesity-related glomerulopathy (ORG) [181]. ORG is a key factor contributing to CKD and is characterized by proteinuria that increases over time in many patients [181]. It differs from nephrotic syndrome by the absence of edema, hypoalbuminemia, and hyperlipidemia [181]. It was revealed that kidneys of children experiencing obesity are larger and exhibit higher renal blood flow compared to those of children of normal weight, suggesting that kidney alterations are induced by obesity in early life [182]. A retrospective cross-sectional study by Marzuillo et al., which was conducted on 2957 children and adolescents affected by obesity, showed an interesting positive correlation between eGFR (estimated glomerular filtration rate) and BMI-SDS and a negative correlation between eGFR and SBP-SDS (systolic blood pressure), HOMA-IR, and duration of obesity [183]. This can be explained by the fact that there is an evolution of obesity-associated nephropathy in which a progressive increase in eGFR levels is observed initially (hyperfiltration), with a subsequent decrease in eGFR levels [183]. In contrast, HOMA-IR increases its effect over the course of the disease [183]. Moreover, a large Israeli study of 1,194,704 adolescents aged 17 years found that adolescents dealing with overweight or obesity presented higher future risk for treated ESRD than those of normal weight [184]. It is unclear what exactly accounts for the association between obesity and CKD; however, hemodynamic (glomerular hyperfiltration, glomerulomegaly, glomerulosclerosis, or increased podocyte injury) and metabolic (adipokine dysregulation, increased insulin resistance, abnormal lipid metabolism, or enhanced oxidative stress) alterations as well as lipid nephrotoxicity (excessive renal fat accumulation) may cause or worsen CKD in individuals affected by obesity [185]. On the other hand, a recent Polish study investigating the link between the function of kidneys and lipid metabolism among children with obesity aged 11–17 years revealed normal renal function in subjects, with a weak association with the parameters of lipid metabolism [186]. Intriguingly, it has also been shown that children of obese mothers are more predisposed to CKD and obesity [185]. 

#### 3.4.7. Gastrointestinal Disturbances

Gastrointestinal diseases such as MAFLD (metabolic dysfunction-associated fatty liver disease, mentioned earlier in the section concerning metabolic complications of obesity) [187], cholelithiasis [188], GERD (gastroesophageal reflux disease) [189], or functional disorders [190] are described in the literature as coexisting with obesity in children.

##### Biliary Duct Diseases

An increasing incidence of gallstones in children has been observed over the last two decades, with a potential cause including a parallel increased prevalence of obesity in the pediatric group [191]. This is an interesting link because cholelithiasis in children has usually been characterized as a rare condition with the main causes including hemolytic diseases, prolonged parenteral nutrition, and prematurity; however, over the last 20 years, the incidence of cholelithiasis unrelated to hemolytic causes has increased from 1.9 to 4% [192]. It has been found that obesity contributes to a higher risk of developing gallstones through excessive liver secretion and bile saturation with cholesterol or impaired cholecyst mobility [188]. Recently, it has also been shown that normal-weight children and adolescents with gallstones and also those who are obese present significantly higher levels of chemerin, an adipokine, than their healthy counterparts [193]. Interestingly, it was also demonstrated that chemerin levels positively correlate with BMI and HOMA-IR in the pediatric population [194]. Hence, the role of chemerin and other adipokines in the development of cholelithiasis needs further investigation. Taken together, children and adolescents whose BMI exceeds the 85th percentile and who experience right upper quadrant or epigastric pain should be suspected of gallbladder disease [195]. A recent multicenter Polish study investigating the relationship between body weight and gallstone disease risk among children revealed that subjects with gallstones presented a substantially higher BMI in comparison to their healthy counterparts [191]. The results of this study are in line with the findings of the Czech study by Frybova et al., which additionally indicated that children with a higher BMI are at a higher risk of developing choledocholithiasis [196]. Furthermore, subjects suffering from choledocholithiasis presented a higher BMI than children with cholelithiasis [196]. As obesity among children increases the likelihood of the occurrence of complications of cholelithiasis, like pancreatitis, and is also associated with a higher rate of hospitalization due to gallstones, it is crucial to reduce the prevalence of obesity in the pediatric population [188]. It should be noted, however, that rapid weight loss by accelerating the elimination of cholesterol that saturates bile has been shown to contribute to the formation of gallstones [197]. The results of an interesting prospective study by Heida et al. showed that 5.9% of children with severe obesity at baseline developed gallstones during a 6-month lifestyle intervention such as exercise or diet [198]. This occurred after losing > 10% of their initial body weight, whereas the highest prevalence of gallstones was observed in the group that lost > 25% of their initial weight [198]. It is worth mentioning that both cholelithiasis and the resulting cholecystitis are important risk factors for gallbladder cancer in later years [199]. The onset of obesity in early adulthood, regardless of prior diagnosis of primary sclerosing cholangitis and cirrhosis, was found to be notably associated with cholangiocarcinoma and may anticipate onset at a younger age [200]. Recently, the influence of obesity on the increased incidence of biliary tract cancer, both with and without previously diagnosed cholelithiasis, has been demonstrated in the adult Asian population [201]. Unfortunately, there is no such information for the pediatric population, which would require further research.

##### Gastroesophageal Reflux 

As mentioned earlier, children with obesity often suffer from gastroesophageal reflux disease (GERD), but they are also at higher risk of esophageal adenocarcinoma, even though they may develop this cancer later in life [189]. Causes of reflux in individuals with obesity include increased intragastric pressure along with lower esophageal sphincter tone and more frequent episodes of its relaxation [202]. A study by Lang et al. found that children affected by obesity and suffering from asthma are more than 7 times more likely to experience multiple symptoms of gastroesophageal reflux (of which the most common were belching, nausea, and swallowing problems) than lean children [203]. In addition, it has also been shown that, among juveniles with obesity, reflux manifestations are associated with worse asthma symptoms, whereas this association was not apparent in the group of their lean counterparts [203]. Further research is needed regarding the association between obesity and GERD in the pediatric population, as specific data on children are rather scarce and most of the literature refers to the adult population.

An interesting correlation was also found between the higher incidence of functional gastrointestinal disorders (FGIDs), such as functional constipation, functional dyspepsia, and irritable bowel syndrome, among children and adolescents struggling with obesity [190]. The mediating role in the link between obesity and the occurrence of functional gastrointestinal disorders may be represented by low fiber intake (which stimulates stool osmolarity), high saturated fat content (which promotes motility), and increased fermentable carbohydrate consumption [204].

#### 3.4.8. Musculoskeletal Disorders

Children and adolescents who are overweight or obese are more likely to develop short- and long-term musculoskeletal disorders, such as pain, injuries, and fractures, due to obesity-induced adverse biomechanical changes in their gait pattern and greater joint burdens [205]. What is more, overweight and obesity occurring in the pediatric population are also associated with impaired postural control, enhanced forces affecting the lower limbs, malalignment of lower extremities, and diminished bone mineral density [206]. The results of the Merder–Coşkun study proved a higher prevalence of musculoskeletal disorders among children dealing with overweight or obesity compared to lean ones [207]. Furthermore, a Polish study investigating the prevalence of postural defects among children and adolescents with excessive body weight found that 69.2% of overweight individuals and 78.6% of individuals with obesity presented postural defects, of which valgus knees and flat feet were the most common [208]. Ferrer et al. demonstrated in their study involving 553 children that body weight has an impact on bone mineralization [209]. The bone mineral content (BMC) and bone mineral density (BMD) measured by bone densitometry (DXA) were highest among those of normal weight and, interestingly, also among those who were overweight [209]. On the other hand, those subjects who were obese or very obese as well as those who were underweight showed lower BMC and BMD values [209]. The increase in BMD and BMC in children dealing with overweight may be the consequence of compensation in response to weight gain; however, this mechanism breaks down among children with obesity, resulting in a decrease in BMD and BMC [209]. Previous studies have demonstrated that children suffering from obesity are more susceptible to extremity fractures, including those in the forearm, and have a higher risk of death due to trauma-related injuries than their non-obese counterparts [210,211]. Musculoskeletal complaints can reduce the determination to exercise and limit the physical capacity of children affected by obesity, potentially contributing to the perpetuation of a sedentary lifestyle and, consequently, obesity [206]. Taken together, it is essential to assess the musculoskeletal system of children who are overweight or obese at every primary care visit, even in the absence of complaints [207]. Intervention aimed at weight loss may help protect them from serious musculoskeletal diseases [207].

#### 3.4.9. Dermatologic Disturbances

As mentioned earlier, obesity is a disease that affects many systems and therefore does not spare the skin either. It has been established that obesity negatively affects the integrity of the skin barrier, alters the structure of collagen, increases the production of sebum and sweat, impairs skin circulation, promotes inflammation, and alters the immune response [212]. Thus, it may contribute to the increased incidence of cutaneous manifestations and disorders, such as acanthosis nigricans, hirsutism, striae distensae, acrochordons, keratosis pilaris, chronic venous insufficiency, plantar hyperkeratosis, cellulitis, psoriasis, skin infections, and hidradenitis suppurativa [213]. Numerous studies regarding the link between obesity and dermatological diseases among adults can be found in the literature; however, evidence for the pediatric population is scarce [214]. Nevertheless, a recent study of 103 children and adolescents with excessive body weight showed a high incidence of skin abnormalities occurring in this group [214]. Skin alterations were associated with increasing BMI and age, and the most prevalent were striae distensae (71%), keratosis pilaris (64.7%), acanthosis nigricans (45%), acne vulgaris (39.2%), acrochordons (25.5%), and plantar hyperkeratosis (17.6%) [214]. In addition, 38.9% of subjects showed reduced dermatological quality of life [214]. Striae distensae are also the most frequently observed dermatologic condition among children with obesity, according to the study by Güven et al. [215]. 

It has also been shown that children and adolescents with hidradenitis suppurativa (HS) have a 2.48 times higher risk of obesity compared to children without HS [216], and, what is more, an increased BMI during childhood is positively correlated with the likelihood of HS occurrence in adulthood [217]. It should also be mentioned that Phan et al. showed an interesting statistically significant association between the incidence of psoriasis in the pediatric population, overweight/obesity, and waist/height ratio > 0.5 [218]. Furthermore, this relationship varies according to the severity of obesity, as patients with moderately severe psoriasis are more likely to be obese in comparison to those with mild psoriasis [218]. A large cross-sectional Israeli study also identified a link between higher BMI and psoriasis among adolescents [219]. 

Moreover, the presence of acanthosis nigricans, keratosis pilaris, and acne vulgaris is associated with increased HOMA-IR (an index of insulin resistance) among children and adolescents with obesity, which may suggest a role for skin lesions as a potential marker of insulin resistance [214]. Insulin resistance has been shown to promote proliferation of fibroblasts and keratinocytes by insulin, insulin-like growth factor receptor-1 (IGFR1), and fibroblast and epidermal growth factors, resulting in the development of acanthosis nigricans [220]. Increased levels of insulin also stimulate androgen production and can reduce serum levels of sex hormone-binding globulin (SHBG), consequently strongly enhancing androgen activity and favoring acne development [221]. Hyperandrogenemia resulting from elevated insulin levels contributes to excessive keratinocyte proliferation of the pilosebacious unit of hair follicles, which may account for the increased incidence of keratosis pilaris in individuals with insulin resistance [222].

## 4. Mental Health Problems in Children with Obesity 

### 4.1. Psychosocial Aspects

Excess body weight, apart from numerous somatic complications, has serious social and psychological consequences, like depressive conditions, eating disorders, attention deficit hyperactivity disorder (ADHD), and low self-esteem. Their prevalence, although difficult to unambiguously determine, seems to be higher than that of typical medical complications. Some specific groups, namely adolescent girls or children suffering from severe and chronic obesity, have been found to be at risk of developing comorbid mental health problems [223]. Depression and eating disorders require the most attention [224]. Anxiety, depression, low self-esteem, and difficulties in emotion regulation can negatively affect obesity treatment.

### 4.2. Depression

Even at a young age, children have a negative attitude toward their obese peers, displaying stigmatization and discrimination. Evidence shows increased vulnerability to depression, anxiety, substance abuse, low self-esteem, and poor body image among children who are teased or bullied because of their weight [225]. Anxiety, self-harm, and suicidal tendencies are also more common among this group of children compared to peers with normal body weight [226]. This association can be attributed to a wide variety of environmental, social, and cultural factors that can also lead to poor outcomes of weight loss interventions [227,228]. Nevertheless, obesity risk factors such as physical inactivity, sedentary lifestyle, unhealthy diet, and excessive screen time have been suggested to be associated with depressive symptoms in adolescents [229]. On the contrary, a healthier lifestyle is associated with a lower risk of depression [229]. Based on current data, approximately 19.44–36.5 million children who are overweight and 12.52–33.11 million children with obesity are diagnosed with major depressive disorder [230].

The multifactorial link between obesity and emotional disturbances is bidirectional and complicated [231]. Several articles have assumed the existence of specific biological factors, in particular a dysregulated hypothalamic–pituitary–adrenal axis with its hyperactivation, exaggerated synthesis/secretion of glucocorticoids [232,233], reduced levels of various neurotransmitters involved in the regulation of the neurological reward system (after chronic exposure to high-fat diet) [234], and higher levels of inflammation cytokines (i.e., C-reactive protein, interleukin-6) [235] as potential pathogeneses of the relationship between depression and obesity. Furthermore, children and adolescents whose mothers experience depression are at greater risk of both depression and obesity [236]. Studies show that the pro-inflammatory consequence of obesity may contribute to the association between weight gain and increased relapse or poor recovery in people receiving treatment for mental illnesses [237,238]. This may suggest that calorie restriction and weight loss diets reduce both inflammation and depressive symptoms in overweight people [239,240]. 

Given the negative health outcomes of depression, systematic screening and effective treatment should be implemented among obese people before adulthood. Moreover, explicit actions like public education, regular exercise, and a balanced diet should be taken to diminish the risk of obesity in the pediatric population.

### 4.3. Eating Disorders

Several mechanisms have been proposed linking obesity with eating disorders. Environmental or individual risk factors for eating disorders (EDs) may also accompany obesity in children and adolescents. Unhealthy eating behaviors, such as emotional eating, skipping meals, or eating close to bedtime, are very common in childhood obesity, which increases the risk of EDs [241]. The most commonly detected EDs in the pediatric population with obesity are bulimia nervosa and binge eating, which are described by poor nutrition and weight control attempts [242]. Nevertheless, anorexia nervosa can also evolve as a result of intensive obesity treatment interventions. EDs can potentially affect almost every organ system, causing severe medical problems that arise from malnutrition, weight changes, or purging (Table 2). Complications of binge eating may include obesity [243].

Children dealing with overweight or obesity often have abnormal eating attitudes and behaviors, which may increase the risk of developing eating disorders in later years. It is estimated that almost a quarter of obese teenagers suffer from binge eating or bulimia. Moreover, adolescent girls with a high BMI and low self-esteem are more likely to start binge eating, have more severe depressive symptoms, and tend to gain more weight [244]. 

Both obesity and eating dysfunctions are usually considered and treated as separate conditions; nonetheless, the bidirectional association of obesity and eating disorders, including eating disorder psychopathology, should be appropriately assessed when planning treatment [242]. Moreover, negative psychological experiences, such as stress, trauma, or stigma, can trigger emotional overeating and lead to a vicious cycle of obesity and depression [245]. 

## 5. Conclusions

Taking into account the consequences of obesity described above, it is obvious that obesity adversely affects the functioning of all systems of the child’s body. This leads to significant complications, not only metabolic but also non-metabolic, which are less obvious. Therefore, obesity should be considered as a serious disorder that significantly decrease the quality of life of children and, with so many complications, can even lead to disability [246]. The rising prevalence of childhood obesity is a serious cause for concern because childhood obesity has been shown to significantly increase the risk of death in early adulthood [247]. As already mentioned, obesity in girls may be associated with serious gynecological and obstetric problems, which may have adverse effects both for them and their future offspring. Therefore, it is extremely important to implement comprehensive actions aimed at stopping the growing epidemic of obesity in the pediatric population and promoting normal body weight in children to protect them against the harmful consequences of obesity. Not only non-pharmacological treatment is important, but also pharmacological treatment of existing complications of obesity and their early detection. It is also necessary to establish global criteria for diagnosing the discussed irregularities, comprehensive actions regarding their prevention and monitoring, and resulting new recommendations regarding both primary and secondary actions.

## 6. Limitations

As our work is a narrative review, we could not rule out bias in the selection of topics covered in the research, but we tried to include as many clinical topics as possible, which makes our review very comprehensive. However, it does not take into account the risk of bias in the assessment of the included studies and is therefore not free from potential errors. There may be some trends, but we tried very hard to assess everything systematically and not exclude works that did not meet our assumptions.

## Figures and Tables

**Table 1:** Obesity complications in children.

Somatic Health Problems			Mental Health Problems
Metabolic	Non-Metabolic		Emotional Disorders
Insulin resistance	**Endocrine**	**Other**	Eating disorders
Diabetes mellitus	Disturbed sexual maturation	CVD	Depression
Impaired glucose metabolism	Hyperandrogenism	Cardiomiopathy	Decreased quality of life
Dyslipidemia	Hirsutism	Asthma	Poor self-esteem
Hypertension	PCOS	Obstructive sleep apnoea	
Metabolic syndrome	Thyroid dysfunction	Allergic diseases	
NAFLD/MAFLD		Pseudotumor cerebri	
		Migraine	
		Oral health disorders	
		Immunologic diseases	
		Chronic kidney disease	
		Functional gastrointestinal disorders	
		Biliary duct diseases	
		GERD	
		Musculoskeletal problems	
		Dermatologic conditions	

**Table 2:** Selected medical complications resulting from eating disorders in children affected by obesity.

EDs Behaviors	Medical Complications
**Bulimia nervosa**	
Fluid and electrolytes	dehydration, hypokalemia, hypochloremia, metabolic alkalosis (vomiting); dehydration, hyperchloremic metabolic acidosis, hypocalcemia (laxative use)
Psychiatric	depressed mood or mood dysregulation; obsessive-compulsive symptoms; anxiety; laxative dependance; suicide
Gastrointestinal	gastroesophageal reflux, esophagitis; Mallory–Weiss tears; esophageal or gastric rupture
Dental	dental erosions
**Binge eating**	obesity with accompanying complications
**Anorexia nervosa**	
Psychiatric	depressed mood or mood dysregulation; obsessive-compulsive symptoms; anxiety; laxative dependance; suicide
Neurologic	cerebral cortical atrophy; cognitive deficits; seizures
Cardiac	decreased cardiac muscle mass, right axis deviation, low cardiac voltage; cardiac dysrhythmias, cardiac conduction delays; mitral valve prolapse; pericardial effusion; congestive heart failure; edema
Gastrointestinal	delayed gastric emptying, slowed gastrointestinal motility, constipation; superior mesenteric artery syndrome; pancreatitis; elevated transaminases; hypercholesterolemia
Endocrinologic	growth retardation; hypogonadotropic hypogonadism: amenorrhea, testicular atrophy, decreased libido; sick euthyroid syndrome; hypoglycemia/hyperglycemia, impaired glucose tolerance; hypercholesterolemia; decreased BMD
Hematologic	leukopenia, anemia, thrombocytopenia, elevated ferritin; depressed erythrocyte sedimentation rate
Fluid and electrolytes	dehydration; hypokalemia, hyponatremia
**Refeeding**	night sweats; polyuria, nocturia; refeeding syndrome: electrolyte abnormalities, edema, seizures, congestive heart failure (rare)

Adapted from Rosen; American Academy of Pediatrics [208].

## Data Availability

No new data were created or analyzed in this study. Data sharing is not applicable to this article.
